# The Burden of Leprosy in Cameroon: Fifteen Years into the Post-elimination Era

**DOI:** 10.1371/journal.pntd.0005012

**Published:** 2016-10-12

**Authors:** Earnest Njih Tabah, Dickson Shey Nsagha, Anne-Cecile Zoung-Kanyi Bissek, Martin W. Bratschi, Theophilus Ngeh Njamnshi, Gerd Plushke, Alfred Kongnyu Njamnshi

**Affiliations:** 1 National Yaws, Leishmaniasis, Leprosy and Buruli ulcer Control Programme, Ministry of Public Health, Yaounde, Cameroon; 2 Swiss Tropical and Public Health Institute, Basel, Switzerland; 3 University of Basel, Basel, Switzerland; 4 Faculty of Medicine and Biomedical Sciences, The University of Yaounde 1, Yaounde, Cameroon; 5 Department of Public Health and Hygiene, Faculty of Health Sciences, The University of Buea, Buea, Cameroon; 6 Division of Operational Research in Health, Ministry of Public Health, Yaounde, Cameroon; 7 School of Health and Medical Sciences, Catholic University of Cameroon, Bamenda; 8 Department of Neurology, Central Hospital Yaounde, Yaounde, Cameroon; Fondation Raoul Follereau, FRANCE

## Abstract

**Background:**

Cameroon achieved the elimination target of leprosy in 2000, and has maintained this status ever since. However, a number of health districts in the country continue to report significant numbers of leprosy cases. The aim of this study was to assess the burden of leprosy in Cameroon from 2000 to 2014.

**Methods:**

We obtained and analysed using the new leprosy burden concept of analysis, leprosy surveillance data collected between 2000 and 2014 from the National Leprosy Control Programme.

**Principal findings:**

Cameroon achieved leprosy elimination in 2000, registering a prevalence rate of 0.94/10,000 population. The prevalence rate dropped further to reach 0.20/10,000 population (78% reduction) in 2014. Similarly, the new case detection rate dropped from 4.88/100,000 population in 2000 to 1.46/100,000 population (85.3% reduction) in 2014. All 10 regions of the country achieved leprosy elimination between 2000 and 2014; however, 10 health districts were still to do so by 2014. The number of high-leprosy-burden regions decreased from 8 in 2000 to 1 in 2014. Seven and two regions were respectively medium and low-burdened at the end of 2014. At the health districts level, 18 remained at the high-leprosy-burdened level in 2014.

**Conclusion:**

The leprosy prevalence and detection rates as well as the overall leprosy burden in Cameroon have dropped significantly between 2000 and 2014. However, a good number of health districts remain high-leprosy-burdened. The National Leprosy Control Programme should focus efforts on these health districts in the next coming years in order to further reduce the burden of leprosy in the country.

## Author Summary

Cameroon achieved the elimination of leprosy in 2000, however, a number of areas in the country continue to report high numbers of cases. We conducted this study to assess the burden of leprosy in Cameroon from 2000 to 2014. We obtained and analysed leprosy data for this period from the National Control Programme. After elimination in 2000, the leprosy prevalence rate continued dropping, to reach 0.20/10,000 population (78% reduction) in 2014. Similarly, the new case detection rate dropped to 1.46/100,000 population (85.3% reduction) in 2014. All 10 regions of Cameroon achieved leprosy elimination by 2014; however, 10 health districts were still to do so. Using the new leprosy burden concept of analysis, the number of high-leprosy-burden regions decreased from 8 in 2000 to 1 in 2014, meanwhile 18 health districts remained high-leprosy-burdened at the end of 2014. In conclusion, leprosy prevalence and detection rates as well as the overall leprosy burden in Cameroon have dropped significantly between 2000 and 2014. However, a good number of health districts remain high-leprosy-burdened. The National Leprosy Control Programme should focus efforts on these health districts in the next coming years in order to further reduce the disease burden in the country.

## Introduction

Leprosy is the oldest disease known to humanity, as recent genomic studies have traced *Mycobacterium leprae*, its causative agent, along human dispersal in the past 100,000 years [1]. It affects peripheral nerves, the skin and mucosa of the upper respiratory pathways [2]. Transmission is believed to be through nasal droplets or prolonged skin contact with an untreated patient, however, the exact mode of transmission is still unclear [3,4]. Untreated patients or those diagnosed late would develop irreversible disabilities and disfiguring complications. These physical complications associated with socio-cultural construction of leprosy are responsible for stigma and social exclusion of victims [5,6].

Effective control of leprosy only began in the 1940s with the discovery of dapson [6,7]. The dapson mono-therapy was replaced by multi-drug therapy (MDT) in the early 1980s [8,7]. Furthermore, a simplified classification as well as treatment regimens for each class was established, so that patients with 1–5 lesions were classified as paucibacillary (PB) leprosy and were treated for six months. Those with 6 or more lesions were classified as multibacillary (MB) and treated for 12–24 months, and subsequently for 12 months [9,10,11].

The results of MDT implementation were very encouraging, with a relapse rate of <1% and a remarkable drop in global leprosy prevalence from 5.37 million registered cases in 1985 to 3.74 million in 1990 [12]. These developments led the World Health Assembly (WHA) to adopt a resolution (WHA 44.9) in 1991, to eliminate leprosy as a public health problem by the year 2000, defining elimination as a leprosy prevalence rate of <1 case per 10,000 population [13].

At the end of 2000, leprosy elimination was achieved globally and in107 countries (including Cameroon) out 122 countries that were considered endemic in 1985 [14,15].

After achievement of leprosy elimination in Cameroon in 2000, the objectives of the National Leprosy Control Programme (NLCP) were focused on consolidating the status at the national level and to further eliminate the disease at sub-national levels. However, some health regions and health districts (HD) have continued to report significant numbers of cases [16]. Furthermore, the declaration of elimination has led to significant reduction in resource allocation for leprosy control activities in the country.

The objective of this study was to assess the leprosy burden in Cameroon in the post-elimination era from 2000–2014, using data from routine reporting available at the NLCP, and to make recommendations for acceleration of its elimination at sub-national levels.

## Materials and Methods

We obtained and analysed routine leprosy surveillance data of 2000 to 2014 from the NLCP. Throughout the period, patients suspected of leprosy were confirmed by establishing the presence of one or more of the following three cardinal signs: hypo-pigmented or reddish skin patch with definite loss of sensation; an enlarged peripheral nerve trunk, with loss of sensation and/or weakness of muscles supplied by that nerve; and the presence of acid-fast bacilli in a slit skin smear on microscopy [11]. A patient case record file (CRF) was opened for each patient and information was also registered in a local facility treatment register (FTR). Patients were classified and treated based of the number of skin lesions as specified above [11]. A positive skin smear classified the patient as MB regardless of the number of skin lesions.

Routinely, data were compiled from FTRs and aggregated into the HD leprosy register (DLR) from which a statistical report was drawn using a standard reporting form and transmitted to the regional level. Each region in turn transmitted on a quarterly basis an aggregate of district reports to the NLCP. Reports from the regions were captured at the national level electronically using Microsoft Excel spread sheets.

For the purpose of this review, annual statistical reports of the ten regions and 181 HD of Cameroon, available at the NLCP were reviewed. The annual statistical reports were summaries of leprosy cases registered each year per HD, providing information for the calculation of the different leprosy elimination indicators [17]. Each regional annual statistical report was cross-checked with HD statistical reports transmitted to the regions. Furthermore, for each region, statistical reports from a sample of HD were compared with data in their respective DLR for quality assurance.

The national leprosy burden analysis was based on the leprosy elimination indicators published by The International Federation of Anti-Leprosy Associations (ILEP) [17]. Accordingly, for each year, the point prevalence and point prevalence rate at the end of the year per 10,000 population, the number of cases detected and new case detection rate (NCDR) per 100,000 population were calculated. Among new cases detected, the MB proportion; the child (0–14 years of age) proportion; the grade 2 disability (G2D) proportion; the G2D rate per 100,000 population; the female proportion; and the point prevalence/new case detection (P/D) ratio were also calculated. Population estimates of regions and HD used for the calculation of indicators were based on the 2005 general population and housing census report of Cameroon [18]. Trend analyses of the key indicators for the period 2000–2014 were done using Microsoft Excel. A linear regression analysis of each indicator curve was performed using the Statistical Package for Social Sciences (SPSS) version 20, and the regression coefficient for each curve was tested for statistical significance in the variation of the curve.

The WHO Regional Office for Africa (Afro) recently developed a new indicator called the “Leprosy Burden Score (LBS)” for assessing leprosy burden at national and sub-national levels (Table 1) [19].

**Table 1:** Scale for the leprosy burden score (LBS) assessment.

Scale	Detection (new cases)		Point prevalence rate per 10,000 population	NCDR per 100,000 population	% MB among new cases	% children among new cases	% G2D among new cases	% females among new cases	P/D ratio	G2D rate per 100,000 population	LBS
	For Regions	For HD									
High	>100 = 2	> 20 = 2	>2 = 2	>20 = 2	<50 = 2	>20 = 2	>20 = 2	<40 = 2	>2 = 2	>1 = 2	≥5 = 2
Medium	21–100 = 1	11–20 = 1	1–2 = 1	10–20 = 1	50–75 = 1	10–20 = 1	10–20 = 1	>60 = 1	1–2 = 1	0.5–1 = 1	3–4 = 1
Low	0–20 = 0	0–10 = 0	<1 = 0	<10 = 0	76–100 = 0	<10 = 0	<10 = 0	40–60 = 0	<1 = 0	<0.5 = 0	0–2 = 0

HD = Health district, NCDR = New case detection rate, MB = Multibacillary, G2D = Grade 2 disability, P/D = Prevalence/Detection, LBS = Leprosy burden score

The LBS is a composite indicator, involving nine leprosy elimination indicators [17]. Depending on the cut-off points (see Table 1), each individual indicator is graded into High = Red (score = 2), Medium = Yellow (score = 1), and Low = Green (score = 0). The sum total of the individual indicator scores, the LBS, is in turn graded into three levels of: High = Red (score = 2) when LBS equal 5 or more, Medium = Yellow (score = 1) when LBS is between 3 and 4, and Low = Green (score = 0) when LBS is 2 and below.

LBS were determined for each region and HD of Cameroon at five-year intervals from the year 2000 (2000, 2005, 2010, and 2014)and used to categorize them into high, medium or low-leprosy-burdened. Regional and HD leprosy burden trend maps from 2000 to 2014 were then established using the ArcGIS software (Environmental Systems Research Institute, Redlands, USA).

### Ethics statement

This study was instructed by the Cameroon Ministry of Public Health Decision N° 0486/D/MINSANTE/CAB and was approved the National Ethics Committee of Cameroon through the authorization N^o^ 172/CNE/SE/2011. All data were anonymized and confidentiality was strictly respected in the data handling and analysis.

### Limitations

There were missing data in some HDs for some of the years under review. A number of report files were damaged and some reports were incompletely filled. However, the proportion of missing data was not significant (about 3%) and did not influence the quality of the review.

## Results

### Leprosy trend in Cameroon from 2000 to2014

Data was available for all the years from 2000 to 2014 except for information on females among new cases, which was available only from 2005. We confirmed that Cameroon achieved leprosy elimination at the end of 2000, recording a point prevalence rate of 0.94/10,000 population.

**Fig 1:**
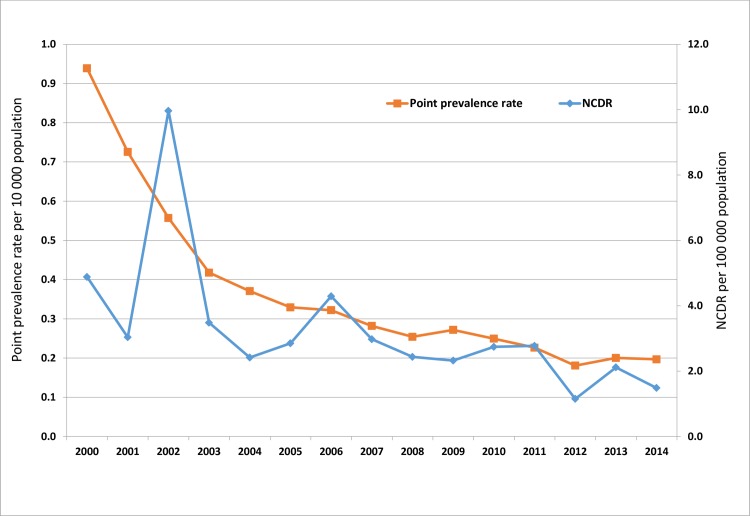
Trends in the leprosy point prevalence rate and NCDR from 2000–2014. The point prevalence rate declined from 0.94/10,000 in 2000 to 0.20/10,000 population in 2014 (P<0.001) accounting for a 78% reduction. Similarly the annual NCDR declined from 4.88/100.000 population in 2000 to 1.46/100.000 population in 2014 (P = 0.018) accounting for an 85.3% reduction. However, two peaks in annual NCDR were noticed in 2002 and 2006 with annual NCDR of 9.96/100,000 and 4.29/100,000 population respectively.

The point prevalence rate declined from 0.94/10,000 in 2000 to 0.20/10,000 population in 2014 (P<0.001) (Table 2 and Fig 1). This decline accounted for 78% reduction in the prevalence rate, with the largest reduction, 64.9%, occurring between 2000 and 2005. From 2006 to 2014, the annual leprosy prevalence rate was rather stagnant. A similar pattern was followed by the leprosy NCDR, with a decline from 4.88/100,000 population in 2000 to 1.46/100,000 population in 2014 (P = 0.018) (Table 2 and Fig 1), accounting for an 85.3% reduction. The largest reduction occurred between 2002 and 2007, followed by a relative stagnation in the NCDR from 2008 to 2014.Two peaks in annual NCDR were however noticed in 2002 and 2006 with annual NCDR of 9.96/100,000 and 4.29/100,000 population respectively.

**Table 2:** Trends in leprosy elimination indicators in Cameroon from 2000–2014.

Year	Population estimate	Registered cases at end of year	Point prevalence rate per 10,000 population	New cases detected	NCDR per 100,000 population	% MB in new Cases	% children in new cases	% female in new cases	% G2D in new cases	G2D rate per 100,000 population
2000	15,137,800	1421	0.94	739	4.88	62	14	ND	9	0.46
2001	15,576,796	1130	0.73	473	3.04	68	11	ND	8	0.24
2002	16,028,524	893	0.56	1597	9.96	64	13	ND	9	0.90
2003	16,493,351	689	0.42	574	3.48	68	13	ND	9	0.30
2004	16,971,658	629	0.37	410	2.42	74	16	ND	5	0.11
2005	17,463,836	575	0.33	498	2.85	70	12	26	7	0.20
2006	17,952,823	578	0.32	770	4.29	75	11	7	6	0.26
2007	18,455,502	520	0.28	549	2.97	70	6	50	3	0.10
2008	18,972,257	482	0.25	462	2.44	79	12	28	5	0.12
2009	19,503,480	530	0.27	453	2.32	75	12	24	4	0.08
2010	19,406,100	484	0.25	532	2.74	76	14	32	5	0.13
2011	19,910,659	451	0.23	552	2.77	53	25	37	6	0.16
2012	20,428,336	369	0.18	235	1.15	76	21	23	7	0.08
2013	20,959,472	420	0.20	443	2.11	86	17	38	8	0.16
2014	21,504,419	426	0.20	315	1.46	87	18	43	7	0.10

NCDR = New case detection rate, MB = Multibacillary, G2D = Grade 2 disability, ND = No data

**Fig 2:**
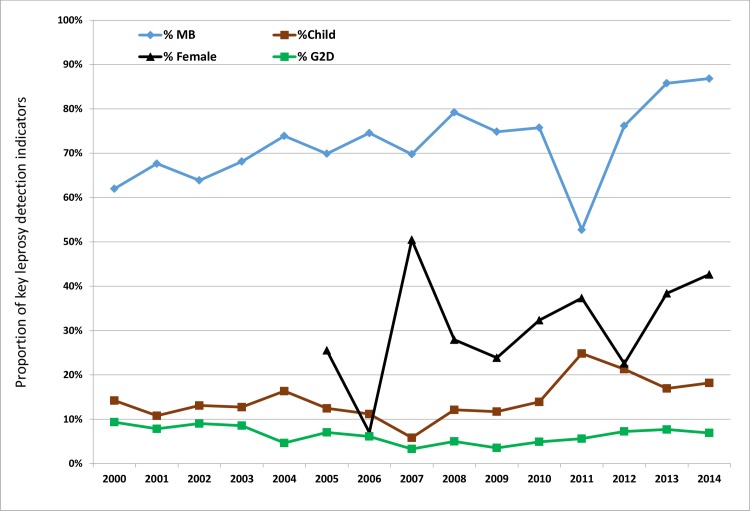
Trends in leprosy detection indicators in Cameroon from 2000 to 2014. The MB proportion was high ranging from 62% to 87%. The G2D proportion remained below 10% throughout the 15-year period. Although the child proportion generally ranged between 10% and 20%, there was a rising tendency from 2008. The Female proportion was fluctuating though with a general rising tendency to reach an acceptable level of 40%.

Among the new cases of leprosy detected, the proportion of MB cases was relatively high throughout the 15-year period investigated, with an increasing trend from 62% in 2000 to 87% in 2014 (P = 0.035). The proportion of child cases generally remained low between 10% and 20% except for the year 2011 when a peak of 25% was observed (P = 0.054). The proportion of new cases with G2D was stable at an average of 6% (P = 0.156). The female proportion was fluctuating, with an overall increasing trend from 26% in 2005 to 43% in 2014 (P = 0.244) (Table 2 and Fig 2).

Between 2000 and 2014, six regions witnessed more than 50% reduction in registered prevalence with the Far-north and the North-west leading with 96% and 86% reduction respectively (Table 3). From 2000 to 2005, six regions namely the Adamawa, East, Far-north, North, North-west and South-west accounted for over 70% of new leprosy case detection in the country. After 2005, four of these regions excluding the Far-north and North-west reported over 70% of new case detection and also registered some of the highest proportions of child and G2D cases in the country (Table 3).

**Table 3:** 5-year trend in selected leprosy elimination indicators by region from 2000 to 2014.

Region	Year 2000				Year 2005				Year 2010				Year 2014				% reduction in registered prevalence between 2000–2014
	New cases detected	Registered prevalence	% Child	% G2D	New cases detected	Registered prevalence	% Child	% G2D	New cases detected	Registered prevalence	% Child	% G2D	New cases detected	Registered prevalence	% Child	% G2D	
Far- north	123	436	19	7	66	65	5	15	21	21	0	14	11	18	0	0	96
North-west	176	203	20	5	28	51	11	0	31	33	3	3	11	28	0	0	86
Littoral	8	154	0	0	38	42	3	5	26	20	0	0	16	23	0	6	85
Centre	88	100	3	18	33	38	0	21	30	33	10	17	19	22	5	16	78
West	52	60	2	35	23	19	4	9	13	22	8	31	24	19	4	0	68
South	10	14	0	0	14	15	7	0	11	12	9	45	6	6	0	0	57
South-west	104	105	14	11	92	112	23	1	144	143	11	3	39	60	13	3	43
East	67	82	21	0	99	82	21	8	71	29	14	3	30	49	30	30	40
Adamawa	29	133	17	3	50	55	6	4	50	51	52	4	73	85	40	1	36
North	82	134	11	9	55	96	15	0	135	120	12	0	86	113	15	8	16
Total Cameroon	739	1421	14	9	498	575	12	7	532	484	14	5	315	423	18	7	70

G2D = Grade 2 disability

### Progress towards the elimination of leprosy at regional and health district levels

**Fig 3:**
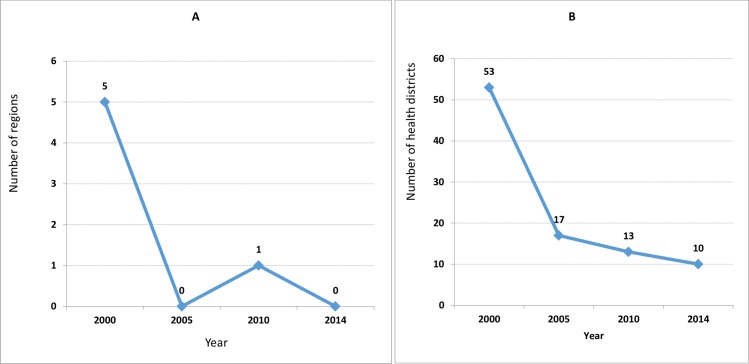
Trends in the number of leprosy endemic regions and health districts in Cameroon from 2000 to 2014. Panel A shows the trend in the number of regions of Cameroon, out of a total of 10 regions, with a point prevalence rate per 10,000 population of more than 1. Panel B shows the trend in the number of HDs of Cameroon, out of a total of 181 HDs, with a point prevalence rate per 10,000 population of more than 1. At the end of 2014, leprosy elimination was achieved in all 10 regions; and is still to be achieved in 10 HDs.

Out of the 10 regions in Cameroon, the number of leprosy endemic regions, with point prevalence rates of 1 or more per 10,000 population, decreased from 5 in 2000 to 0 in 2014 (Fig 3A) meanwhile the number of endemic HDs decreased from 53 to 10 over the same period (Fig 3B). Table 4 lists the remaining 10 leprosy endemic HDs in Cameroon.

**Table 4:** Health Districts which remained hyper endemic for leprosy at the end of 2014.

Region	Health District	Point prevalence rate per 10.000 population
Adamawa	Ngaoundere Rural	2.29
East	Abong Mbang	2.21
	Garoua Boulai	1.05
North	Poli	9.40
North-west	Benakuma	2.38
South-west	Nguti	3.65
	Mundemba	3.25
	Ekondo Titi	1.36
	Akwaya	1.25
West	Galim	1.21

### Progress towards the target of the “Enhanced global strategy for further reduction of leprosy burden (2011–2015)” in Cameroon

**Fig 4:**
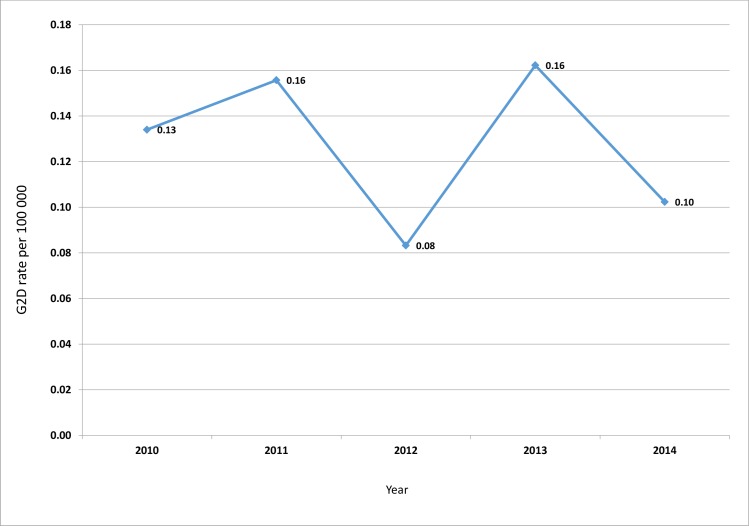
Trend in G2D rate per 100.000 population. The G2D rate decreased slightly from 0.133/100.000 population in 2010 to 0.105/100.000 population in 2014, constituting a 21% reduction.

At the national level the trend in G2D rate decreased slightly from 0.133/100.000 population in 2010 to 0.105/100.000 population in 2014 (P = 0.747) (Fig 4), constituting a drop of 21%.

### Trend in leprosy burden with-respect-to the new Leprosy Burden Score

**Fig 5:**
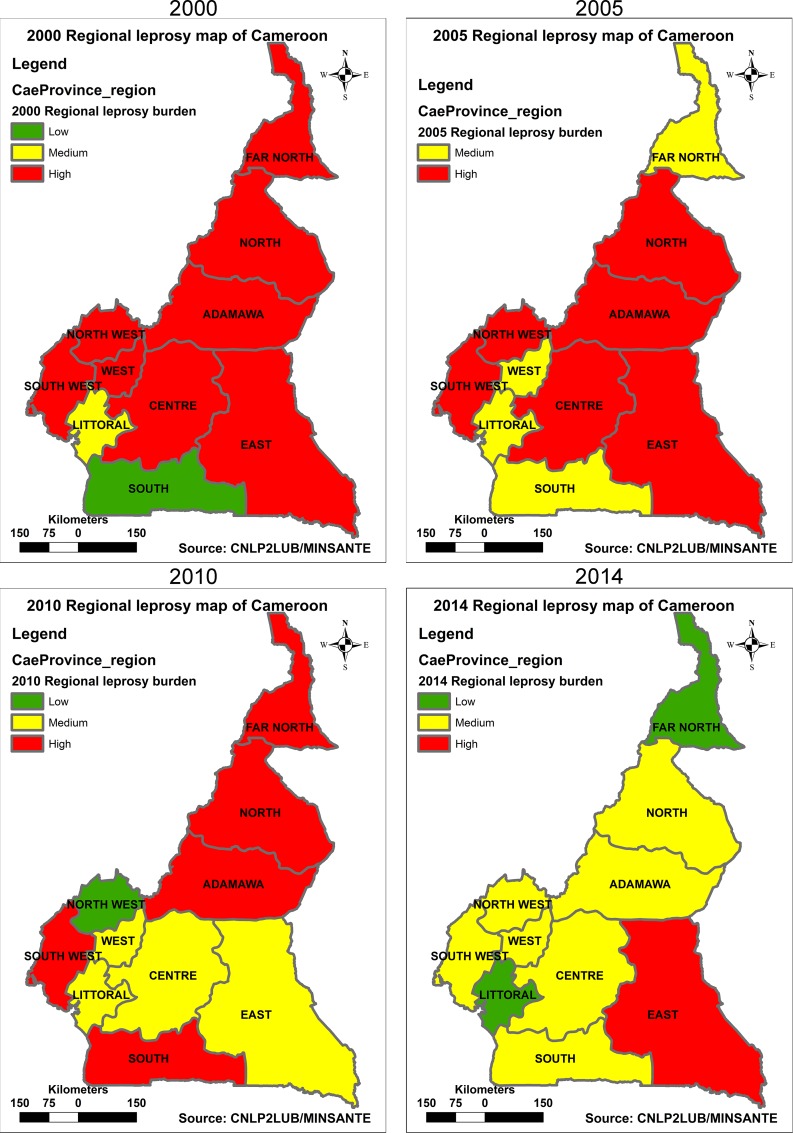
Five-year-interval trend in leprosy burden map of Cameroon by region from 2000 to 2014. In 2000, 8 out of 10 regions in Cameroon were high-leprosy-burdened and one medium-burdened. By 2005 this number decreased to 6 and then to 5 in 2010, and further to 1 in 2014.

A 5-year-interval trend analysis of leprosy burden by region (Fig 5), revealed that in the year 2000, eight regions were high-leprosy-burdened and one medium-burdened. By 2005 the number of high-burdened regions decreased to 6 and then to 5 in 2010, and further to 1 in 2014.

**Fig 6:**
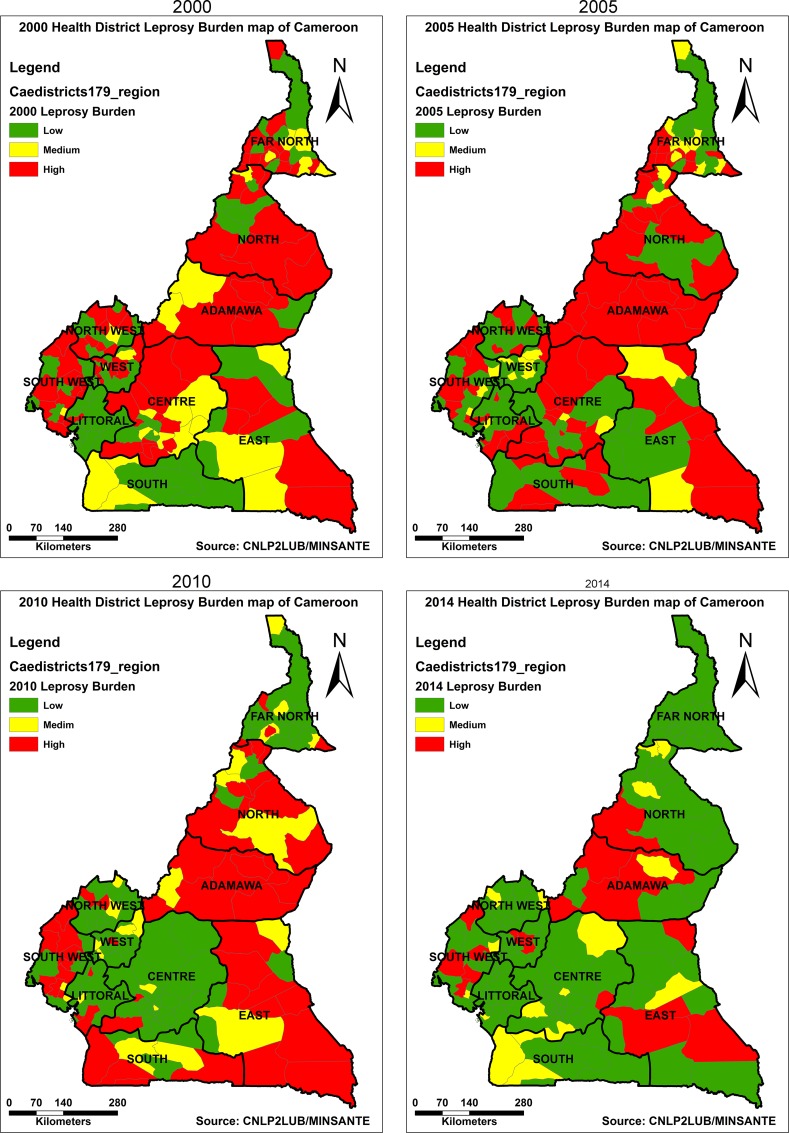
Five-year-interval trend in leprosy burden map of Cameroon by health district from 2000 to 2014. The number of high-leprosy-burdened districts dropped from 68 in 2000 to 18 in 2014. During the same period, the number of medium-burdened districts also witnessed a drop from 31 to 20. The reduction in the number of both high and medium-burdened health districts was gained by low-leprosy-burdened districts that rose from 82 in 2000 to 143 in 2014.

At the HD level, (Fig 6) the number of high-leprosy-burdened districts stagnated at 68 and 69 between 2000 and 2005, and then dropped to 49 in 2010 and further to 18 in 2014. During the same period, the number of medium-burdened districts also witnessed a drop from 31 in 2000, to 20 in 2014. The decrease in the number of both high and medium-burdened districts was gained by low-leprosy-burdened districts that rose from 82 in 2000 to 143 in 2014.

## Discussion

In 1991, The WHA passed a resolution to eliminate leprosy as a public health problem by the year 2000. The yard stick for measuring elimination was the attainment by countries of a leprosy prevalence rate below 1 per 10 000 population [13]. At the end of 2000, the WHO announced the elimination of leprosy as a public health problem globally and in 107 out of 122 countries that were considered endemic in 1985 [14]. Despite elimination at the national level, leprosy has remained a public health problem at sub-national levels in many countries [7,20], and many other concerns were unresolved [21,22].

Cameroon achieved leprosy elimination at the end of 2000 [23]at the national level and has maintained this status to date. The results of our analyses show trends in the prevalence and detection rates. These declined sharply between 2000 and 2007 and then became rather stagnant from 2008 to 2014. These trends match with those of the WHO African Region, where the stagnation was explained by significant increase in case detection in six countries. [19]. The stagnation in the prevalence and new case detection in Cameroon is attributed to high numbers of new leprosy cases being detected in four regions namely the Adamawa, East, North, and South-west(Table 3).

Generally, leprosy transmission in Cameroon between 2000 and 2014 has been moderate considering that the proportion of children among new cases ranged from 10% to 20% over this period. However, high transmission occurred in the Adamawa, East and South-west regions where child proportions above 20% were often registered (Table 3). The proportion of G2D among new leprosy cases was as desired, below 10% throughout the period under review. This places Cameroon in the low-disability-burdened countries [19]. The proportion of females among new leprosy cases has gradually risen to attain an acceptable level of more than 40% implying that more and more affected women are getting access to leprosy care [19]. The high and rising proportion of MB among new leprosy cases could be perpetuating transmission of the disease in the population, as MB cases constitute the main source of infection [24,25,26] (Fig 2). The reduced number of skilled personnel in leprosy diagnosis at the primary health care level, and the passive mode of leprosy case detection in Cameroon may explain the delayed diagnosis and consequently, high MB proportion. There is need for training of operational level staff, as well as the reinforcement of leprosy courses in the curricular of training schools for health personnel and faculties of medicine in the country.

After the achievement of leprosy elimination at national level in 2000 efforts were focused towards eliminating at sub-national levels. Five out of 10 regions were still endemic with a prevalence rate above 1 per 10.000 population in 2000 (Fig 3A). This number dropped to one in 2010, with the Southwest being the only region with a prevalence of 1 per 10.000 population. By 2014 all the regions achieved elimination. With regards to HDs (Fig 2B), those that were still to eliminate leprosy, the number dropped from 53 in 2000 to 10 in 2014. The remaining high endemic HDs at the end of 2014 were concentrated in the Southwest, East, Adamawa, North and West regions (Table 4). Poli HD in the North region was the most endemic in the whole country, with a prevalence rate of 9.40 per 10.000 population in 2014, followed by Mundemba and Nguti in the South-west, with 3.25 and 3.65 per 10.000 population respectively.

The pattern in leprosy prevalence reduction between 2000 and 2014 varied across the regions, sometimes with adjacent regions witnessing totally different patterns. For instance, the Far-north region had a 6-fold reduction in leprosy prevalence compared to the adjacent North region, and the North-west a 2-fold reduction compared to the Southwest region (Table 3). These disparities could be attributed to two major reasons: first, the update of leprosy registers in all ten regions following recommendations of the third meeting of the Technical Advisory Group on Leprosy Elimination [27], allowed the removal of patients unduly maintained in leprosy registers between 2001 and 2004. The highest numbers of such patients were removed from registers in the Far-north and North-west regions. Second, geo-cultural reasons could be responsible for patterns in the North and the South-west region. An active leprosy case finding by the NLCP in Poli HD, detected most of the cases from among the Koma people [28] who reside in the enclaves of the Atlantika Mountains on the border between Cameroon and Nigeria. These are indigenous people with a peculiar lifestyle (little or no proper clothing, poor personal skin hygiene, and probably overcrowding) that may be favourable for the transmission of leprosy. In the South-west region, most of the reported cases come from Akwaya, Ekondotiti, Mundemba, Mbonge, Nguti HDs which are very enclaved HD and where some tribal people believe leprosy is a spell and can only be handled by traditional healers. These beliefs may affect health-seeking behaviours and consequently contribute to the high burdens recorded in these areas.

In launching the “Enhanced Global Strategy for Further Reducing the Disease Burden due to Leprosy (2011–2015)” [29], a new indicator, the G2D rate per 100.000 population, was introduced for monitoring progress in the implementation of the strategy. A targeted 35% reduction in G2D rate by 2015 was set, taking as baseline the rate in 2010 [29]. Between 2010 and 2014, Cameroon achieved a 21% reduction in the G2D rate (Fig 3) below the desired level.

With the achievement of leprosy elimination by all countries, the WHO recently developed a new method for the assessment of leprosy burden [19], and its use for categorising countries and sub national levels as high, medium or low leprosy burdened. The new leprosy burden assessment takes into account not only the prevalence rate but also eight other indicators (Table 1). Using this concept to categorise regions and HDs and to evaluate the leprosy burden trend from 2000 to 2014, we concluded that a lot of progress has been made in reducing leprosy burden at both regional and HD levels in Cameroon (Figs 5 and 6). In 2000, despite elimination of leprosy at national level, eight regions were still highly burdened with leprosy. The situation improved significantly, as by 2014 only one region remained high-leprosy-burdened. Three regions namely East, North-west and South were unstable in their progression from high towards low-leprosy burden. This is probably explained by the degradation of leprosy services in these regions, following the attrition of resources for leprosy elimination activities (Fig 5). Despite the apparent improvement in the reduction of leprosy burden, the NLCP must work hard to bring all the regions to low-burdened, given that seven regions remain medium-leprosy-burdened.

The leprosy burden analysis at the HD level in this study has led to the identification of specific hotspots within the regions (Fig 6) although there has also been a lot of improvement at this level. The number of high-leprosy-burdened HD has dropped from 68 in 2000 to 18 in 2014 (Fig 6). The 18 remaining high-burdened HD are mainly concentrated in the Adamawa, East, North and South-west Regions of the country. These are areas where the NLCP should focus efforts in the coming years, to further optimize leprosy control in Cameroon.

Despite the positive results registered in Cameroon in the post leprosy elimination era, further reduction of leprosy burden in the country is facing huge challenges. After the achievement of leprosy elimination at national level in 2002, government and partner NGO commitments gradually faded away. There has been a reduction in financial and technical support from partners since 2005 to almost complete withdrawal in 2012. For this reason, the NLCP performance has been sub-optimal in essential components such as effective integration of MDT services into the primary health care, regular supervision, community-based surveillance, capacity building and prevention of disability as required by the post elimination WHO strategies [29,30,31].

The WHO lunched a roadmap for accelerating work to overcome the global impact of NTDs for the period 2012–2020 [32]. This roadmap was further endorsed in 2013 by the 66^th^ WHA [33] and the 63^rd^ WHO Regional Committee for Africa (WHO Afro) [34] respectively. Of the 17 NTDs considered in the roadmap, leprosy is being targeted for global elimination by 2020. The roadmap and subsequent WHA and WHO Afro resolutions urge national programmes, member states and support partners to more commitment so that NTD control efforts are sustained and set targets met by 2020.

### Conclusions

In Cameroon, the leprosy prevalence and detection rates have dropped significantly since 2000 but have been stagnating in the last years. Furthermore, the new concept of determining the leprosy burden by using the leprosy burden score, has unmasked problem areas that could not be determine by the prevalence rates alone and revealed alarming disparities of the total leprosy burden at sub-national levels. Thus, eighteen HDs of Cameroon have remained with a high leprosy burden in 2014 despite the long acquired elimination status by the country. The NLCP should focus efforts on these HDs while monitoring the 20 medium burdened HDs as well. With improved government funding and more partner support, the NLCP objectives and the WHO targets can be met in all health districts of Cameroon by 2020.

## Supporting Information

S1 File Compressed shape files _Cameroon health district. The Cameroon health district shape files used for drawing up of the maps in this article were obtained from the Sub Department for epidemiological surveillance in the Ministry of Public Health, Cameroon.
(ZIP)

S1 Dataset Dataset for results presented in the article. (XLSX)

S2 Dataset Dataset for regional leprosy burden maps (Fig 5). (XLSX)

S3 Dataset Dataset for health district leprosy burden maps (Fig 6). (XLSX)
