# Correction: The Burden of Leprosy in Cameroon: Fifteen Years into the Post-elimination Era

**DOI:** 10.1371/journal.pntd.0005305

**Published:** 2017-01-18

**Authors:** Earnest Njih Tabah, Dickson Shey Nsagha, Anne-Cecile Zoung-Kanyi Bissek, Martin W. Bratschi, Theophilus Ngeh Njamnshi, Gerd Pluschke, Alfred Kongnyu Njamnshi

The sixth author's name is spelled incorrectly. The correct name is: Gerd Pluschke. The correct citation is: Tabah EN, Nsagha DS, Bissek ACZK, Bratschi MW, Njamnshi TN, Pluschke G, et al. (2016) The Burden of Leprosy in Cameroon: Fifteen Years into the Post-elimination Era. PLoS Negl Trop Dis 10(10): e0005012. doi: 10.1371/journal.pntd.0005012
